# The impact of maternal care and blood glucose availability on the cortisol stress response in fasted women

**DOI:** 10.1007/s00702-021-02350-y

**Published:** 2021-05-12

**Authors:** Ulrike U. Bentele, Maria Meier, Annika B. E. Benz, Bernadette F. Denk, Stephanie J. Dimitroff, Jens C. Pruessner, Eva Unternaehrer

**Affiliations:** 1 Department of Psychology, Division of Neuropsychology, University of Constance, Constance, Germany; 2 Centre for the Advanced Study of Collective Behaviour, University of Constance, Constance, Germany; 3 Child- and Adolescent Research Department, Psychiatric University Hospitals Basel (UPK), University of Basel, Basel, Switzerland

**Keywords:** Stress, Maternal care, Glucose, Cortisol, Early life stress

## Abstract

Individuals with a history of low maternal care (MC) frequently present a blunted, yet sometimes also show an increased cortisol stress response. Fasted individuals with low blood glucose levels who are exposed to acute stress typically show an attenuated response pattern in this endocrine marker. Despite well-documented metabolic dysregulations after low MC, a possible interaction of both factors has not been investigated yet. Here, we examined the effects of MC and blood glucose concentration on various aspects of the stress response. Fasted women (*N* = 122, mean_age_ = 22.12, sd_age_ = 2.56) who experienced either very high, high, or low MC (based on the Parental Bonding Instrument) were randomly assigned to consume grape juice (condition *sugar*), or water (condition *water*) prior to being exposed to the Trier-Social-Stress-Test for groups. Salivary cortisol and alpha amylase, blood glucose, and mood ratings were assessed repeatedly. Using multilevel mixed models, we replicated the boosting effect of glucose on the cortisol stress response. While we found neither an effect of MC, nor an interaction between MC and blood glucose availability on the cortisol stress response, we observed an effect of MC on the amylase stress response. We discuss the results in the light of links between various stress/energy systems that possibly mediate health-related MC effects.

**Supplementary Information:**

The online version contains supplementary material available at 10.1007/s00702-021-02350-y.

## Introduction

Acute threat activates the hypothalamic–pituitary–adrenal (HPA) axis and the sympathetic adrenomedullary (SAM) system (Andrews et al. 2013; Chrousos 2009). These two stress systems mediate adaptive cardiovascular and metabolic processes (Sapolsky et al. 2000) that are essential for human survival. As such, a robust stress response is important to maintain health. In contrast, dysfunctional adaptations of the stress systems have been associated with an increased risk for the development of a variety of physical and mental diseases (Chrousos 2009). 

One prominent factor that can trigger such long-lasting endocrine dysregulations is an adverse environment during childhood and adolescence, or early life adversity (ELA) (Fogelman and Canli 2018). ELA includes severe traumatic experiences like abuse or neglect, but also more subtle experiences (Bugental et al. 2003) like a dysfunctional parent–child relationship with a low extent of parental care (Engert et al. 2010; Felitti et al. 1998; Ali and Pruessner 2012). In the last decades, it has been shown that a history of ELA is associated with dysregulations of the cortisol stress response up into adulthood. While some studies report increased cortisol stress responses (Rao et al. 2008), the majority of studies report blunted cortisol stress responses following ELA (Bunea et al. 2017). This blunted pattern further seems to be influenced by the severity, and/or frequency of the experienced ELAs (Engert et al. 2010; Voellmin et al. 2015). For example, Engert et al. (2010) found that individuals reporting low maternal care during childhood and adolescence showed a diminished cortisol stress response compared with individuals reporting high maternal care (MC). In contrast, Pruessner et al. (2004) identified increased cortisol stress responses in individuals reporting low MC. These findings suggest that a low extent of MC is associated with HPA-axis dysregulations, but findings regarding the direction of these dysregulations are still inconclusive. Here, other moderating factors might contribute to the partially inconsistent results.

One such moderating factor regulating HPA-axis (re-)activity could be energy availability (Iranmanesh et al. 2011; Kirschbaum et al. 1997). Here, energy availability refers to the accessibility of substrates in blood circulation which serve to supply the organism with energy. Both low energy availability [e.g., after fasting (Akana et al. 1994)] and high energy availability [e.g. through food intake after fasting periods (Kirschbaum et al. 1997)] influenced the reactivity of the HPA axis in past studies. For example, male participants with low euglycemic blood glucose concentration after fasting did not show an increase in cortisol levels in response to acute psychosocial stress, while participants consuming glucose before stressor onset exhibited a robust cortisol stress response (Kirschbaum et al. 1997). This restoring effect of energy intake was further shown to be exclusive for glucose, since neither fat nor protein intake triggered a similar response (Gonzalez-Bono et al. 2002). Even participants who fasted for a shorter time period (no food intake within the last 3-4 h) showed increased cortisol levels in response to acute stress after the consumption of glucose-containing drinks. Thus, findings indicate that the consumption of glucose-containing drinks facilitates a robust cortisol response in comparison with participants who fasted for 3 h prior to the experiment (Zänkert et al. 2020) and in comparison with participants who consumed non-caloric sweetener, or water after 4 h of fasting (von Dawans et al. 2020). Taken together, while the mechanism of the observed boosting effect of glucose on the endocrine stress response is still unclear, processes that are triggered by the ingestion of glucose seem to be key drivers (Kirschbaum et al. 1997).

Although these findings indicate that both childhood experiences of MC and blood glucose availability are involved in the regulation of HPA-axis reactivity, no study has yet investigated the interaction between these two factors. Since such an interaction could possibly explain the inconsistent findings on the effects of MC on cortisol stress reactivity, we wanted to provide and test a theoretical framework on the interplay between ELA, blood glucose availability, and cortisol stress reactivity. Specifically, we propose that high blood glucose availability through sugar intake might serve as a strategy by which subjects who experienced ELA could compensate for an otherwise blunted cortisol reactivity. This framework is based on diverse theoretical considerations and empirical findings: first of all, individuals with a history of ELA exhibit an increased risk for a variety of metabolic diseases [e.g., diabetes and obesity (Danese and Tan 2014; Felitti et al. 1998)] which can partially be explained by higher food intake (Greenfield and Marks 2009), resulting in higher glucose availability and supply (Peters et al. 2011). Second, the cortisol stress response predicts the amount of food intake after stress: participants with a blunted cortisol response show higher food intake after a stressor (Wingenfeld et al. 2017). Finally, indirect empirical support can be derived from studies investigating the effects of chronic stress (similar to ELA), the cortisol stress response and food intake. Specifically, chronically stressed individuals with blunted cortisol stress responses (Tomiyama et al. 2011) showed higher intake of sugar-containing food after psychosocial stress (Tryon et al. 2013), indicating a possible compensation strategy to normalize cortisol reactivity.

The aim of this study was thus to test whether glucose consumption can act as a compensatory strategy for individuals with a history of mild ELA, operationalized by a lower extent of MC, to normalize their (commonly blunted) cortisol stress response. For this, we invited individuals with very high, high, or low levels of perceived MC to the laboratory after an overnight fast of at least 8 h. After the consumption of either water (condition *water*) or grape juice (condition *sugar*), participants were exposed to a psychosocial stress test. Throughout the experiment, physiological (salivary cortisol and alpha amylase) and psychological (subjective pleasure and arousal) stress responses, as well as blood glucose levels were measured repeatedly. First, we hypothesized that the consumed drink (sugar or water) would have an effect on the cortisol stress response, such that higher cortisol responses would be observed after sugar in comparison to water ingestion. Second, we hypothesized that the extent of MC (very high, high, and low) would be related to the cortisol stress response. Specifically, a lower extent of MC should be related to more blunted cortisol responses, as low MC indicates maternal neglect, which has often been conceptualized as one type of ELA in previous research (Smith and Pollak 2020). Third, we hypothesized that the extent of MC (very high, high, and low) and the consumed drink (sugar, or water) would interact, such that the effect of MC on the cortisol stress response would be modulated by glucose availability.

## Methods

### Participants

Recruitment took place at the University of Constance. Before being invited to the laboratory, all participants completed an online eligibility screening. Exclusion criteria were: (1) an age younger than 18, or older than 30 years (to control for the effect of age on HPA-axis activity; similar age ranges have been used in Kirschbaum et al. 1997; Engert et al. 2010), (2) current pregnancy (Mastorakos and Ilias 2003), (3) symptoms of moderate-to-severe depression [indicated by a Beck’s Depression Inventory (BDI) II sum score > 18; (Beck et al. 1996; Kirschbaum et al. 1999)], (4) being under- or obese [indicated by a body mass index (BMI) < 17.5 or > 30; Gwirtsman et al. 1989; Kumari et al. 2010), (5) smoking > 5 cigarettes per day, (6) working night-shifts, (7) a history of brain damage, (8) current drug or medication intake affecting the autonomous, endocrine, or central nervous system (Carpenter et al. 2011), and (9) reported physical or psychiatric illness. If not otherwise stated, criteria were assessed via self-report using single items concerning the respective criterium. Finally, due to a very low number of eligible male participants (10% of the recruited sample after 6 months of testing], we decided to study females only.

To assure equal distribution of participants in the very high, high, and low MC groups (*n* = 40 in each group), the extent of MC was determined in the online screening using the Parental Bonding Instrument (PBI; Parker et al. 1979) that retrospectively assesses the child’s perception of parental care and overprotection in the first 16 years of life. The PBI consists of 25 items, which are rated for each parent from the child’s perspective. For each parent, two subscale scores are computed (*care*, 12 items, and *overprotection,* 13 items). Items are rated on a 4-point Likert scale ranging from 0 (“very unlikely”) to 3 (“very likely*”*). Higher scores indicate a higher degree of perceived care, and a higher degree of perceived overprotection, respectively. In this study, the maternal care subscale of the PBI was used to assess the extent of MC as a mild form of ELA, with either low MC (MC scores < 27), high MC (MC scores ≥ 27, but < 33), or very high MC (MC scores ≥ 33).

*N* = 122 eligible women (mean_age_ = 22.12, sd_age_ = 2.56) participated in the study. The study was approved by the Ethics Committee of the University of Constance, Germany, prior to its conductance (IRB statement 12/2017), and was carried out in accordance with the ethical standards of the Declaration of Helsinki. All participants gave written informed consent prior to participation and received financial compensation (€25) or research credit hours (2.5 h).

### Procedure

**Fig. 1:**
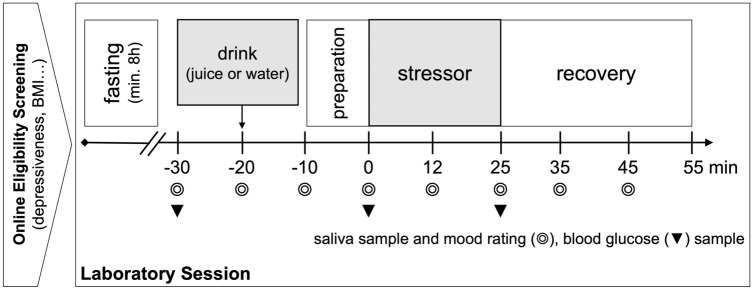
Overview of the study procedure: The experimental manipulation involved drinking 400 ml of water, or 400 ml of grape juice (containing 64 g of glucose), stress was induced using a modified version of the Trier Social Stress Test for Groups (TSST-G)

Laboratory sessions were scheduled to start at either 8 or 10 a.m. to control for circadian rhythms in cortisol secretion (Kudielka et al. 2004) and to facilitate the fasting period for participants. The sessions lasted approximately 2 h and participants were invited to the laboratory in groups of up to four. Participants were asked to refrain from alcohol, coffee, sugar-containing beverages, and smoking 1 h prior to the laboratory session. In addition, participants were instructed to not engage in heavy physical activity 1 day before testing. Participants were requested to fast for at least 8 h prior to the laboratory session. The study procedure is shown in Fig. 1.

After participants arrived in the laboratory, a baseline of physiological and mood measures was obtained (see below). Participants were then randomly assigned to the experimental conditions and consumed either 400 ml of grape juice containing 64 g of sugar (in the following referred to as experimental condition *sugar*, *n* = 61), or 400 ml of water (control condition *water*, *n* = 61). Consequently, our design resulted in six different groups: low MC and sugar (*n* = 21), low MC and water (*n* = 21), high MC and sugar (*n* = 20), high MC and water (*n* = 20), very high MC and sugar (*n* = 20), and very high MC and water (*n* = 20). Subsequently, participants completed a set of questionnaires. During the following stress phase, participants were exposed to a modified version of the Trier Social Stress Test for Groups (TSST-G; von Dawans et al. 2011), a standardized laboratory stressor for group settings. During the final recovery phase, participants completed questionnaires and were further allowed to relax. Salivary samples for subsequent cortisol and alpha amylase analyses were obtained repeatedly using the Salivette devices (Sarstedt, Nümbrecht, Germany). Alongside, participants rated their mood using the Affect Grid (Russell et al. 1989). Furthermore, blood glucose levels were measured using capillary blood. At the end, participants were debriefed and compensated for participation.

### Stress induction

Ten minutes after drink consumption, participants were exposed to a modified version of the Trier Social Stress Test for groups (TSST-G; von Dawans et al. 2011), which constitutes an efficient procedure to induce psychosocial stress in a group setting with six participants (Dickerson and Kemeny 2004; Kirschbaum et al. 1993). The standardized protocol consists of a fictive job interview during which a free speech and a difficult arithmetic task have to be performed in front of an expert committee. Due to feasibility, the TSST-G was modified to be suitable for groups of two to four, a procedure which has successfully been used and reported in related studies of our group (Meier et al. 2020, August 14). After a preparation phase (10 min), participants were introduced to a two-member, mixed-sex committee in front of which they performed their two tasks (24 min with 3 min per task per participant). During both tasks, participants were videotaped and called in random order.

### Stress measures

#### Physiological measures

Salivary samples for biochemical cortisol (nmol/l) and alpha amylase (U/ml) analysis were gathered using Salivettes (Sarstedt, Nümbrecht, Germany) at eight predefined timepoints (see Fig. 1). The first sample was taken prior to the acclimatization to the laboratory session and served to familiarize subjects with the sampling procedure. It was therefore excluded from statistical analyses. The samples were stored at − 20 °C until study completion and sent to the biochemical laboratory of the University of Trier for biochemical assays. For the detection of cortisol, the samples were analyzed using a time-resolved immunoassay with fluorometric detection (DELFIA) with an intra-assay coefficient of variation (CV) below 6.7% according to the manufacturer (Dressendörfer et al. 1992). In our sample, the inter-assay CV was below 14.4% (mean = 4.2%). For the detection of alpha amylase, the enzyme kinetic method was used (alpha-Amylase Saliva assay, RE80111, IBL International GmbH, Hamburg, Germany) with an intra-assay CV below 6.9% according to the manufacturer. The inter-assay CV in the analysis conducted in Trier was below 15.7% in all but four samples, which were determined in duplicates a second time due to a very high inter-assay CV (> 20%) in the first duplicate determination.

At three prescheduled timepoints (see Fig. 1), blood glucose levels (mg/dl) were obtained from capillary blood of the fingertip using disposable lancets (Roche Diabetes Care, Mannheim, Germany) and a glucometer (A. MENARINI diagnostics, Berlin, Germany).

#### Subjective measures

The Affect Grid (Russell et al. 1989) was used to measure subjective stress along the two orthogonal, bipolar dimensions pleasure, and arousal. The dimension pleasure ranges from 1 (“unpleasant”) to 9 (“pleasant”); the dimension arousal ranges from 1 (“sleepy”) to 9 (“highly aroused”). Higher scores indicate a high degree of pleasure and arousal. The Affect Grid has been shown to have adequate reliability and validity, and is assumed to be specifically beneficial for repeated-measures designs (Russell et al. 1989). Subjective stress ratings were obtained simultaneously to the saliva samples.

#### Data processing

For cortisol, individual trajectories were screened visually to check for non-responders. As some participants were expected to show reduced or even absent cortisol stress responses, we could not use an *increase criterion* as proposed by Miller et al. (2013) to define cortisol non-responders. Visual exploration of individual cortisol trajectories indicated *n* = 18 participants with the first two cortisol values exceeding 20 nmol/l and continuously decreasing levels over the course of the experiment. Since experimental sessions started early (either at 8 or 10 a.m.), these high initial values were interpreted as being caused by the cortisol awakening response (CAR, Pruessner et al. 1997), which would interfere with the ability to create a consecutive cortisol stress response due to a ceiling effect (Kudielka et al. 2004). Non-responders were more frequently found in the 8 a.m. group, regardless of condition. Therefore, these participants were excluded from the following statistical analyses leading to a final sample size of *N* = 100, and the following six experimental groups: low MC & sugar (*n* = 18), low MC and water (*n* = 16), high MC and sugar (*n* = 18), high MC and water (*n* = 15), very high MC and sugar (*n* = 15), and very high MC and water (*n* = 18). The approach can be retraced online (https://osf.io/bcu2a/).

Next, biological markers (cortisol, alpha amylase, and blood glucose) were screened for missing values. While there were no missing values in cortisol and alpha amylase, three missing blood glucose values from *n* = 3 participants in the water control group were imputed linearly. In a second step, values which deviated more than 3SD from the grand mean were replaced by the respective 3SD value of the corresponding biological marker.

Since session start had a significant effect on cortisol levels with overall higher cortisol values found in the 8 a.m. in comparison to the 10 a.m. session, cortisol values were further z-standardized within each of the two groups. By that, we aimed at making the trajectories of the two groups more comparable with each other, while at the same time keeping model parsimony low.

As a common measure of stress reactivity, we used the area under the curve with respect to increase (AUCi; Pruessner et al. 2003). We calculated AUCi’s for cortisol (AUCi_cort_), alpha amylase (AUCi_amy_), and subjective stress (AUCi_stress_) trajectories from 0 min to + 45 min, and for glucose (AUCi_glucs_) from − 30 to + 25 min.

### Statistical analysis

Analyses were conducted using R (version 4.0.2), including the packages “nlme”, “ez”, “psych”, “pastecs”, “lsmeans”, “pgirmess”, “gmodels”, and “RVAideMemoire”. A *p* value < 0.05 was considered significant. Normal distribution was tested using Shapiro–Wilk tests, homogeneity of variance was tested using Levene’s tests. Potential multicollinearity in predictors was investigated using variance inflation factor (vif < 10). Model residuals were examined for normality using QQ-plots.

#### Preliminary analysis

Depending on data properties and fulfillment of test assumptions, we first tested for differences between the six groups (low MC and sugar, low MC and water, high MC and sugar, high MC and water, very high MC and sugar, very high MC and water) in the potential covariates age, body mass index (BMI), BDI and Rosenberg Self-Esteem Scale (RSES) scores, session start, TSST group size, and hormonal status (variable with three levels: follicular phase, luteal phase, oral contraceptives use) using Analyses of Variance (ANOVAs), Kruskal–Wallis Rank Sum Test, or Pearson’s Chi-Squared test.

Next, we conducted a manipulation check to examine whether blood glucose concentration increased in participants consuming grape juice. We further tested whether levels of subjective stress increased in response to the TSST-G. Manipulation checks were conducted using growth curve models (see below).

#### Confirmatory analysis

For our confirmatory analysis with the dependent variable cortisol, we decided to focus on the period after stressor onset (from 0 to + 45 min, in the following referred to as stress-response period). Effects of stress are typically reported using approaches like the analysis of (a) changes is stress measures via repeated measures such as growth curves, or (b) overall stress *reactivity* using cumulative indices such as the AUCi. We here decided to report the results of both approaches in parallel.

To test our hypotheses, we examined the factors Drink (2 levels: water = 0, sugar = 1), MC (3 levels: 1 = very high MC, 2 = high MC, 3 = low MC), and their interaction as predictors for (a) changes in cortisol levels over time during the stress-response period using growth curve models, and (b) overall cortisol stress *reactivity* using an ANOVA with AUCi_cort_ as dependent variable.

To test the first hypothesis that the HPA stress response is increased after sugar load, we examined whether *Drink* is a significant predictor of (a) changes in cortisol levels during the stress-response period (indicated by a significant *Time* × *Drink* interaction in the growth curve approach)*,* and (b) overall cortisol stress *reactivity* (AUCi_cort_ indicated by a main effect of *Drink* in the ANOVA approach). The same procedure was used to test the second hypothesis that HPA stress responses are blunted after exposure to low maternal care using the predictor *MC* instead of *Drink*. To test the third hypothesis that there is an interaction between both factors, we examined whether the *Drink* × *MC* interaction is a significant predictor of (a) changes in physiological stress indices during the stress-response period (indicated by a significant three-way *Time* × *Drink* × *MC* interaction in the growth curve approach), and (b) overall stress reactivity (AUCi_cort_ indicated by a significant *Drink* × *MC* interaction in the ANOVA approach).

The growth curve models to analyze physiological stress trajectories over time were built hierarchically based on best fit using the following steps: (1) random effects for intercept; (2) trend over time (linear, quadratic, cubic); (3) random slopes; (4) autoregressive order 1 correlation structure (CAR1), which is commonly assumed for repeated measure models with continuous time-intervals; (5) adding predictors and their interaction. The model that was most likely generating the observed data was identified using log-likelihood ratio tests. After we had defined the model with the best fit, we examined the effects of our predictors according to our hypotheses. Variables that were unevenly distributed across the six groups were included as covariates (see preliminary analysis).

#### Exploratory analysis

Being exploratory, we further examined whether *Drink*, or their interaction would predict the change over time in both blood glucose concentrations, and alpha amylase, as an index of the SAM system, using the same procedure as described above (growth curves).

## Results

### Preliminary analyses

Overall, we implemented a 2 [Drink: glucose, water] × 3 [MC: very high, high, low] between subject design. We found no difference between the six groups in age, BMI, RSES, session start, TSST group size, or hormonal status (all *p* > 0.05). However, we found group differences in BDI (K-W Chi-squared (5) = 22.06; *p* < 0.001), which was driven by differences between the *MC* groups (K–W Chi-squared (2) = 18.87; *p* < 0.001), but not the *Drink* groups (K–W Chi-squared (1) = 0.84; *p* = 0.359). Thus, we decided to evaluate the effect of BDI in our analysis by estimating the effects of interest both including and excluding BDI as a covariate. Detailed information on participant characteristics can be found in the supplementary material (Table S1).

**Fig. 2:**
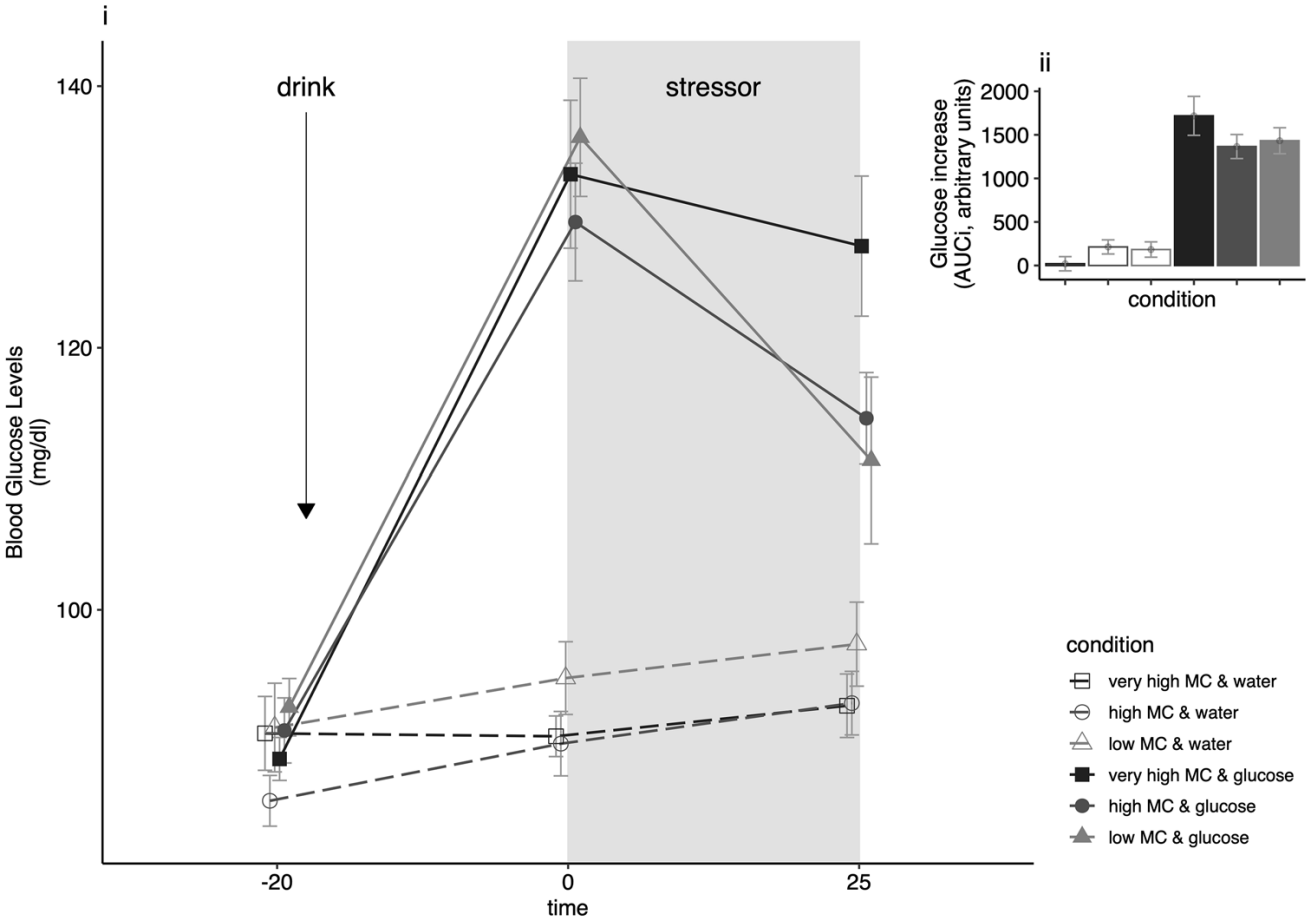
Changes in blood glucose levels over time (i), and in response to the drink (ii) per experimental condition (values are depicted as mean ± SE)

Growth curve analysis of the three blood glucose assessments (timepoints: − 30, 0, + 25 min) revealed that the data were best explained by a quadratic trend, and a random intercept and random slopes model (supplementary material, Table S2). *Drink* showed a main effect (F(1, 98) = 37.37, p < 0.001) and an interaction with *Time*^2^ (*F*(2, 196) = 79.63, *p* < 0.001) to predict blood glucose concentration. Figure 2 shows blood glucose changes per group over time.

Growth curve analysis of pleasure and arousal assessments (timepoints: − 20, − 10, 0, + 12, + 25, + 35, + 45 min) suggested that the data were best explained by a quadratic trend with random intercept and random slopes, and a first-order autoregressive covariance structure (CAR1) for both subjective stress variables (supplementary material, Table S3 and S4). In all six groups, pleasure decreased from baseline to TSST and then increased again during the recovery period. This was paralleled by a self-reported increase in arousal from baseline to TSST, which decreased during the recovery period.

### Confirmatory analyses

The best model for cortisol trajectories during the stress-response period indicated a cubic trend for time, and random effects for intercept (Intra-Class Correlation Coefficient = 0.71) and slopes (supplementary material Table S5). The model considering the CAR1 covariance structure did not converge, and therefore had to be omitted. The model included all 500 observations of all 100 participants.

#### Cortisol stress trajectories

**Fig. 3:**
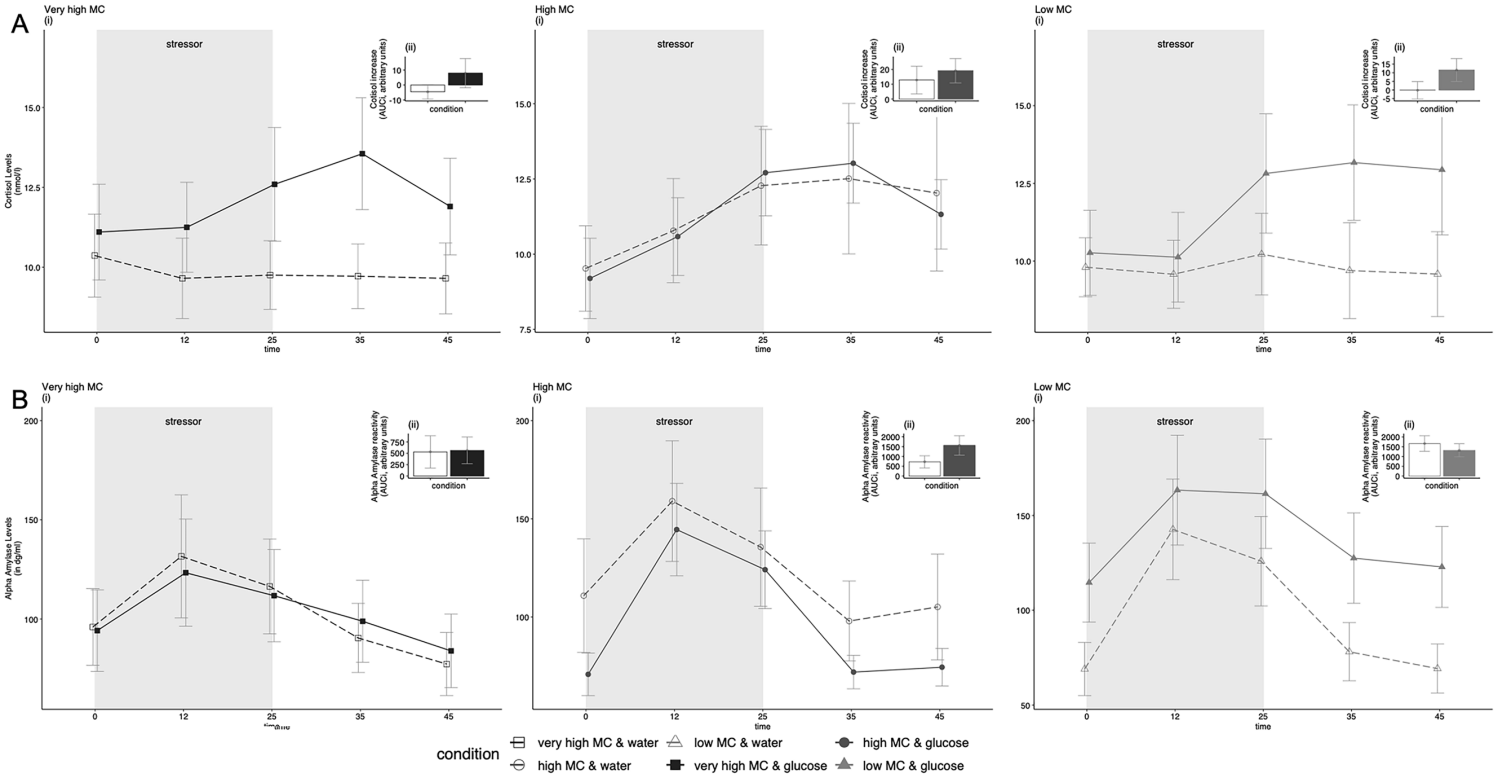
Cortisol (**a**) and alpha amylase (**b**) stress trajectories over time (i) and stress reactivity in response to the stressor (by means of the area under the response curve; AUCi) in the very high, high, and low maternal care (MC) groups per experimental condition (glucose, water) (values are depicted as mean ± SE): looking at cortisol trajectories (**a**, i), we found a significant Time^3^ × Drink interaction (*p* = 0.012), but no support for a *Time*^3^ × *Drink* × *MC* interaction; for alpha amylase trajectories (**b**, i), we found a significant *Drink* × *MC* interaction (*p* = 0.003)

The model including the *Time*^*3*^ x *Drink* interaction (hypothesis 1) improved the model fit significantly (supplementary material, Table S5) compared with the models not including the interaction term. Accordingly, we found support for our hypothesis that the change in cortisol levels over time depended on *Drink*. Participants in the sugar condition showed a cortisol increase during and post stressor, while participants in the water condition displayed stable trajectories in cortisol across the stress and recovery period (Fig. 3a). A summary of the *Time*^3^ × *Drink* interaction model excluding covariates is provided in Table 1, and regression coefficients can be found in the supplementary material (Table S6).

**Table 1:** Summary of final model with Time (linear, quadratic, and cubic) and Drink as predictors and z-standardized cortisol levels during the stress-response period as outcome variable

Effects	Statistic	*p*
Intercept	*F*(1, 394) = 1.02	0.314
Time^3^	*F*(3, 394) = 10.26	< 0.001
Drink	*F*(1, 98) = 2.09	0.152
Time^3^ × Drink	*F*(3, 394) = 3.72	0.012

Effects of the final Time^3^ × Drink growth curve model for z-standardized cortisol trajectories. Time^3^ represents the cubic effect of time. Time^3^ × Drink represents the interaction between the cubic effect of time and Drink. Exclusion of data values with residuals > 3SD or inclusion of BDI as a covariate did not change the significance of the results

Next, we did not find a significant improvement in model fit when including *MC* (very high, high, and low) as fixed effect, or the *Time*^3^ × *MC* interaction as predictors for cortisol concentration (hypothesis 2; supplementary material, Table S5). Thus, we had to reject the hypothesis that MC influences cortisol stress responses and did not evaluate the fixed effects of these models.

Finally, inclusion of the interaction term *Time*^3^ × *Drink* × *MC* did also not increase model fit compared with the models including lower order interaction terms (hypothesis 3; supplementary material, Table S5). Thus, we had to reject the hypothesis that the interaction between blood glucose availability and MC predicts the changes in cortisol concentration and we did not evaluate any effects of this model.

In all models, including BDI score as a covariate or excluding data values with residuals > 3SD did not change the significance of the results. A summary of the model including all hypothesized predictors is provided in the supplementary material (Table S7).

#### Cortisol stress reactivity

AUCi_cort_ was normally distributed for all subgroups, except the group with high MC in the water condition (*p* < 0.001). Natural log transformation of (AUCi_cort_ + 70) made distribution normal across all but one (high MC and water) groups. Levene’s test revealed no violation of the variance homogeneity assumption (*F*(5, 94) = 0.93; *p* = 0.470). Thus, we used ANOVA (type III, type II) to examine group differences in AUCi_cort_ based on *Drink*, *MC* and their interaction. The *Drink* x *MC* interaction was not significant (*F*(2, 94) = 0.07; *p* = 0.932, partial eta^2^ = 0.001) and we did not find a main effect for *Drink* (*F*(1, 94) = 2.07; *p* = 0.154; partial eta^2^ = 0.026) or for *MC* (*F*(2, 94) = 2.19; *p* = 0.118; partial eta^2^ = 0.044). Exclusion of three values with residuals > 3SD or inclusion of BDI as a covariate did not change interpretation of the results.

### Exploratory analysis

#### Exploring whether blood glucose response after drink consumption depends on MC

With respect to blood glucose, previously described analyses were conducted with blood glucose concentration as the dependent variable. Details on model comparison are provided in supplement material (Table S2).

The final model revealed a significant *Time*^2^ × *Drink* × *MC* interaction. In the sugar condition, the decrease in blood glucose depended on MC group, with those participants reporting very high MC having the slowest glucose metabolism, and those in the low MC group having the fastest. A summary of the *Time*^2^ × *Drink* × *MC* model is provided in Table 2. Regression coefficients are provided in the supplement material (Table S8).

**Table 2:** Summary of final model with Time (linear, quadratic), Drink, and MC as predictors and blood glucose levels as outcome variable

Effects	Statistic	*p*
Intercept	*F*(1, 188) = 10,242.98	< 0.001
Time^2^	*F*(2, 188) = 113.01	< 0.001
Drink	*F*(1, 94) = 36.44	< 0.001
MC	*F*(2, 94) = 1.28	0.283
Time^2^ × Drink	*F*(2, 188) = 78.87	< 0.001
Time^2^ × MC	*F*(4, 188) = 1.26	0.288
Drink × MC	*F*(2, 94) = 0.33	0.721
Time^2^ × Drink × MC	*F*(4, 188) = 2.83	0.026

Effects of the final Time^2^ × Drink × MC growth curve model for blood glucose trajectories. Time^2^ represents the cubic effect of time. Time^2^ × Drink represents the interaction between the quadratic effect of time and Drink. Time^2^ × MC represents the interaction between the quadratic effect of time and MC. Drink × MC represents the interaction between Drink and MC. Time^2^ × Drink x MC represents the three-way interaction between the quadratic effect of time, Drink, and MC. Correcting for BDI score did not change the results. When data values with residuals > 3SD were excluded, Time^2^ × Drink × MC lost significance, but remained significant at trend level (*F*(2,189) = 2.92; *p* = 0.056)

In all models, correcting for BDI score did not change the results. When we excluded data values with residuals > 3SD, the three-way interaction in the model with blood glucose as dependent variable lost significance, with (*F*(2,189) = 2.92; *p* = 0.056).

#### Exploring whether Drink, MC, and their interaction influence the alpha amylase stress response

We ran the same analyses as described previously with salivary alpha amylase measured during the stress-response period as the outcome variable. The best model indicated a cubic trend for time, and random effects for intercept and slopes. The model considering the CAR1 covariance structure did not converge, and therefore had to be omitted. The model included all 500 observations of all 100 participants. Details on model comparison are provided in supplementary material (Table S9).

The final model did not include a significant *Time*^3^ × *Drink* or *Time*^3^ × *MC* interaction, suggesting that the change in alpha amylase over time did neither depend on *Drink*, nor on *MC*. However, there was a significant *Drink* × *MC* interaction. This interaction is illustrated in Fig. 3b showing no difference between drink conditions in the very high MC group, while in participants with high MC, the water group showed higher alpha amylase concentration during all stages of the TSST and recovery period as compared with the sugar group; in contrast, participants with low MC who consumed sugar generally showed higher alpha amylase concentrations compared with the low MC water group. Further adding a *Time*^3^ × *Drink* × *MC* interaction term did not improve model fit (supplementary material, Table S9). Correction for BDI as a covariate or exclusion of data values with residuals > 3SD did not change interpretation of the results. A summary of the final *Drink* × *MC* model is provided in Table 3. Regression coefficients can be found in the supplementary material (Table S10).

**Table 3:** Summary of final model with Time (linear, quadratic, cubic), Drink, and MC as predictors and alpha amylase levels during stress as outcome variable

Effects	Statistic	*p*
Intercept	*F*(1, 388) = 89.78	< 0.001
Time^3^	*F*(3, 388) = 28.31	< 0.001
Drink	*F*(1, 94) = 0.72	0.398
MC	*F*(2, 94) = 0.36	0.702
Time^3^ × Drink	*F*(3, 388) = 0.70	0.551
Time^3^ × MC	*F*(6, 388) = 1.90	0.080
Drink × MC	*F*(2, 94) = 6.11	0.003

Effects of the final Drink × MC growth curve model for alpha amylase trajectories. Time^3^ represents the cubic effect of time. Time^3^ × Drink represents the interaction between the cubic effect of time and Drink. Time^3^ × MC represents the interaction between the cubic effect of time and MC. Drink × MC represents the interaction between Drink and MC. Correction for BDI as a covariate or exclusion of data values with residuals > 3SD did not change interpretation of the results

## Discussion

The aim of this study was to investigate whether the consumption of glucose versus water affects the endocrine stress response in fasted women and whether this effect would depend on differing extent of childhood MC. We found that the effects of consumed drink and MC on the stress response differed depending on the biological stress marker, and on how these markers were analyzed (trajectory vs. reactivity). Specifically, we found an effect of sugar consumption on cortisol trajectories, indicating increased cortisol responses during stress after ingestion of sugar compared with water (hypothesis 1). However, there was neither a significant effect of MC on cortisol trajectories (hypothesis 2), nor did the effect of sugar consumption on the cortisol trajectory depend on MC (hypothesis 3). Regarding cortisol reactivity (by means of the AUCi), we did neither find main effects nor an interaction effect of sugar consumption and MC (hypothesis 2).

Findings of our exploratory analysis suggest that the effect of sugar consumption on alpha amylase trajectories was dependent on MC. While participants with very high MC showed comparable trajectories in alpha amylase after stress in both experimental conditions, participants in the high and low MC group showed differences in alpha amylase depending on the drink they consumed: in the high MC group, those in the glucose condition showed lower levels of alpha amylase, compared with those in the water condition, while the opposite pattern emerged in the low MC group. Finally, participants reporting very high MC showed a slower return to baseline in blood glucose concentration after glucose consumption compared with participants reporting low MC, who showed the fastest decline.

With regard to our first hypothesis, our results confirmed that glucose ingestion prior to stress increased the cortisol stress response after long fasting periods. These results expand former findings from samples of exclusively male participants (Gonzalez-Bono et al. 2002; Kirschbaum et al. 1997). They are also in line with findings regarding the effect of glucose consumption prior to stress after shorter fasting intervals (von Dawans et al. 2020; Zänkert et al. 2020). So far, it seems that this boosting effect of glucose on the cortisol stress response after longer fasting intervals is similar across sexes. One previous study that examined the cortisol stress response after a short fasting interval of 3 h explored the effect of sex (Zänkert et al. 2020) and, interestingly, observed no differences in the cortisol stress response in women after juice consumption in comparison to no drink. Since fasting per se influences cortisol levels (Dallman et al. 2004), the different fasting durations [overnight fast of at least 8 h in our study vs. 3 h (Zänkert et al. 2020)] may explain this discrepancy between findings (Kasckow et al. 2001). Yet, future studies could test potential sex differences in the context of longer fasting periods more rigorously by implementing a sex-balanced design.

In general, our results confirm the strong mutual connections between cerebral centers responsible for glucose regulation and the regulation of the HPA axis (Rohleder and Kirschbaum 2007). However, the exact underlying mechanisms still remain unclear. The selfish brain theory might provide one potential explanation (Peters 2011; Peters and Langemann 2009; Peters et al. 2004). This theory states that the brain—the body’s primary glucose consumer—has the capacity to control the distribution of glucose within the organism via the activation of the HPA-axis, whereby it selfishly guarantees its own energy supply: When blood glucose availability is high, the pancreas releases insulin to facilitate the absorption of glucose into peripheral tissue. The brain then triggers the release of cortisol to suppress further insulin-induced absorption to peripheral tissue, preventing blood concentration from becoming too low.

Yet, it is noteworthy that our results did not reveal a corresponding effect of blood glucose availability on cortisol reactivity (AUCi) (Gonzalez-Bono et al. 2002). This diverging finding is in line with a previous study, in which male participants fasted for 4 h and consumed either sugar, artificial sweetener, or water prior to stress exposure (von Dawans et al. 2020). Since former studies examining the effect of longer fasting intervals did not consistently include the analysis of cumulative reactivity measures like the AUCi, it is difficult to compare our finding to these previous studies (Kirschbaum et al. 1997). While both methodological approaches—change over time and reactivity in response to stress—seem to be inherently linked to each other (and former studies reporting both measures often report that findings resemble each other, e.g., Pruessner 1998), they contain distinct pieces of information: on patterns that occurred over time (growth curves), and on overall intensity of the reaction (AUCi). The current results indicate that different methodological approaches yield different results and reporting them alongside might provide a more comprehensive picture of biological system integrity.

Contrary to our second hypothesis, the results did not show that the low, high, and very high MC groups differ in the displayed stress-induced trajectories and cortisol stress reactivity, with several factors potentially at play here. First, although a history of ELA has repeatedly been linked to HPA-axis dysregulations in general, results regarding the direction of these dysregulations are still inconsistent, indicating either blunted (Bunea et al. 2017), increased (Rao et al. 2008), or even no clear differences in cortisol responses following ELA (DeSantis et al. 2011). This inconsistency might be due to variations in the form of ELAs these studies have focused on: a blunted cortisol stress response has predominantly been found after severe forms of ELA, like childhood maltreatment (Carpenter et al. 2011; Voellmin et al. 2015; Elzinga et al. 2008). Maltreatment, which might be mainly characterized by event-related stressors such as exposure to physical, emotional, or sexual violence, represents the presence of a negative event. However, low maternal care might have a somewhat different nature, since here a primary care person does not provide enough or stable nurturing responses and thus represents the absence of positive events, stability, and/or safety (Smith and Pollak 2020). Both, maltreatment and low parental care are often co-occurring, but not always. Thus, both blunted and increased cortisol responses have been reported after low MC (Pruessner et al. 2004; Engert et al. 2010), maybe because the presence or absence of maltreatment have not been considered. Because we investigated a healthy, low-risk sample, we operationalized ELA by the extent of MC, which commonly shows more variation in these populations.

Second, fasting per se resulting in a low energetic status affects HPA-axis regulation (Dallman et al. 2004). Previous studies that have reported blunted cortisol stress responses following (both severe and more subtle forms of) ELA did not report participants’ energetic status, and probably tested participants with euglycemic blood glucose levels (e.g., after a brief fast of up to 3 h). In contrast, in the current study participants were required to fast for at least 8 h, resulting in blood glucose concentration in the low euglycemic range. Since postingestive processes triggered by meal consumption only last a few hours, the implementation of a long fasting period allowed us to make sure that postingestive effects triggered by the last ingested meal were already decayed at the time of testing. Therefore, it is possible that the strong effect of fasting might have masked the effect of MC as a form of ELA on HPA-axis regulation. In sum, our study could not confirm an effect of a history of mild ELA on different measures of the cortisol stress responses.

The effect of sugar consumption on both cortisol trajectory and reactivity to stress did not differ between the low, high, and very high MC groups (hypothesis 3). This could imply that blood glucose availability facilitates HPA-axis stress responses independent of ELA which implicates that individuals with low MC could indeed “profit” from higher blood glucose availability when attempting to modulate these stress responses (albeit they do not profit more than, e.g., participants with a high or very high MC history). As such, future research should investigate whether individuals with a history of ELA increase their sugar consumption in comparison to individuals with lower ELA to modulate stress.

Results from our exploratory analyses revealed that the extent of MC predicted the speed by which blood glucose levels returned back to baseline after glucose consumption and stress. Participants reporting very high MC showed the slowest glucose metabolism, while participants reporting low MC displayed the fastest. This is an interesting finding, although we did not anticipate it and as such it is a post hoc observation. Interestingly, this is paralleled by findings, indicating that perinatal stress is related to increased levels of insulin in a population with high risk of psychosis (Perry et al. 2021), and that childhood trauma is linked to increased insulin levels in patients with first episode psychosis (Tosato et al 2020). As such, low MC could lead to higher insulin levels which would be reflected in faster glucose metabolism, as indicated by our findings. Be that as it may, our finding points to an additional effect of MC which could also point to a possible explanation for lower cortisol levels in subjects with MC; if they generally have a higher glucose metabolism, they might more frequently be tested for their cortisol responses when low in glucose and thus present with blunted cortisol responses. As far as the reason for a faster glucose metabolism is concerned, we can only speculate on the molecular mechanisms underlying such an association. One explanation could be that the extent of MC programs epigenetic patterns of the glucocorticoid receptor (GR) (Tyrka et al. 2012), also in the periphery, which in turn regulates the availability of glucose transporter proteins (Kyrou and Tsigos 2009; Sapolsky et al. 2000). These plasma membrane proteins regulate blood glucose concentration by facilitating the uptake of glucose from blood into peripheral tissue. These differences could be interpreted as a sign of systematic adaptation of metabolic systems that are involved in glucose and energy storage in people with ELA. If the experience of a stressful and insecure environment in early ages leads to a perception of the world as threatening and uncertain (e.g., about the continuous unavailability of resources), this may lead to energy being stored more efficiently to have it available at a later time. Also, this could lead to a greater motivation and lower skill to inhibit the desire to eat sugary food, that is effortlessly accessible in the industrialized world (Peters et al., 2017). Therefore, the adult situation prompts a mismatch between anticipated and actual environment for individuals with ELA (Nederhof and Schmidt 2012; Schmidt 2011), resulting in a situation that could inherit a higher risk for health conditions.

Finally, further exploratory analyses revealed that the effect of glucose availability on SAM system activity depended on MC, whereby participants with high MC showed higher, and participants with low MC showed lower levels of alpha amylase when glucose was available. This finding implies that it is rather the association between glucose availability and the activation of the SAM system (Peters et al. 2011), than the HPA-axis that is affected by a history of low MC. The possibly diverging effects on the different stress-response systems (Ali and Pruessner 2012) in combination with the availability of glucose need to be investigated more systematically in future studies, but could also point to metabolic effects impacting on the autonomous nervous system.

There are some limitations to this study. First, the generalizability of the presented sample is limited to young, well-educated, healthy women. While we initially planned to implement a sex-balanced design, we hit on intractable challenges in the recruitment of men (approximately 70 females and 7 males recruited after 6 months); thus, we decided to focus on females in this project. Our study adds to findings in male participants by presenting findings from an exclusively female sample. However, the interaction between MC and glucose availability should further be examined in men. Second, some of the results are based on exploratory analyses and thus warrant replication in an independent sample. Third, exclusion criteria were mainly measured using single items concerning the respective criterium (e.g., psychiatric illness) that were answered via self-report. As answers might be biased for various reasons (e.g., social desirability, memory effects), the use of more objective or validated measures would contribute to a more reliable assessment of exclusion criteria in future studies. Fourth, while we invited participants in the morning hours (at 8 or 10 a.m.) to decrease the burden of the 8 h fast, we did not assess the time of awakening. During the processing of the cortisol trajectories, we realized that a substantial part of participants displayed very high initial cortisol baseline values (> 20 nmol/l), especially in the 8 a.m. group, suggesting that participants might have still been inside the first hour after awakening, which might have prevented a cortisol stress response to occur. We addressed this issue by excluding potential non-responders and using a z-standardization that aimed at making the cortisol trajectories of the two groups (8 versus 10 a.m.) more comparable with each other, while at the same time keeping model parsimony low. It would have obviously been preferable to avoid testing within the first hour after awakening. Finally, we cannot rule out the potential effect of salivary flow rate on the salivary cortisol and alpha amylase assessments, since we did not assess it. There is an ongoing debate on whether one should control for salivary flow rate (Strahler et al. 2017), and if, and how it is affected by different saliva assessment approaches (i.e., passive drool method, Salivette devices, etc.). Although some studies found no effect of salivary flow rate on increases in alpha amylase after stress (Rohleder et al. 2006), it could be advisable to assess and control for salivary flow rate until this discussion is completely settled (Strahler et al. 2017).

Apart from these limitations, our study did have some strengths, such as (a) the randomized allocation of participants with different extent of MC to the experimental groups, (b) the inclusion of different objective markers of the biological stress systems, (c) a powerful statistical approach (growth curve modeling), and (d) an exclusively female sample to replicate and extend previous findings from male samples (e.g., Kirschbaum et al. 1997). While we considered menstrual cycle or oral contraceptive use, we did not include this potential covariate in our analyses, because there were no differences between MC and Drink group with regard to these variables. Notably, this is one of few studies that tried to examine and compare different methodological approaches and different biological stress markers. Indeed, we found that the effects of the predictors strongly depended on the methodological approaches and biological marker investigated. In terms of content, our results convey a complex, but also more global picture of the interplay of the stress response and the metabolic system. In light of the rise in metabolic and stress-related disorders, researchers should further target to understand mechanisms behind increased risks for subgroups of individuals such as people with a history of low MC or other forms of childhood adversity. On the long run, we think that this approach has high potential to reduce (a) financial costs for health authorities, and, more importantly, (b) the impairing physical and psychological consequences of such diseases on suffering individuals.

## Supplementary Information

Below is the link to the electronic supplementary material.

Supplementary file1 (PDF 147 KB)

## Data Availability

Supplementary information is available online at https://osf.io/3y9ed/ (Open Science Framework project https://doi.org/10.17605/OSF.IO/NWMV6). A preprint of this manuscript has been published on PsyArXiv (https://psyarxiv.com/axkde; https://doi.org/10.31234/osf.io/axkde).

## Code availability

Code is available online at https://osf.io/3y9ed/ (Open Science Framework project https://doi.org/10.17605/OSF.IO/NWMV6).
