# Supplementary material for: The impact of maternal care and blood glucose availability on the cortisol stress response in fasted women
Source: J Neural Transm (Vienna). 2021 May 12;128(9):1287–300. doi: 10.1007/s00702-021-02350-y (PMC8423636; doi:10.1007/s00702-021-02350-y)
Supplement: Supplementary file 1 — Supplementary file1 (PDF 147 KB) [file 702_2021_2350_MOESM1_ESM.pdf]

Supplementary Material

The impact of maternal care and blood glucose availability on the cortisol stress response in fasted women

Ulrike U. Bentele*[1], Maria Meier*[1], Annika B. E. Benz[1], Bernadette Denk[1,2], Stephanie Dimitroff[1], Jens C. Pruessner[1,2], Eva Unternaehrer[1,3]

***Shared first authorship**

[1]*Department of Psychology, Division of Neuropsychology, University of Constance, Constance, Germany*
[2]*Centre for the Advanced Study of Collective Behaviour, University of Constance, Constance, Germany*
[3]*Child- and Adolescent Research Department, Psychiatric University Hospitals Basel (UPK), University of Basel, Switzerland*

*Corresponding author:
Maria Meier (maria.meier@uni-konstanz.de)

ORCIDs:

Ulrike U. Bentele	https://orcid.org/0000-0002-3121-1957
Maria Meier	https://orcid.org/0000-0002-1655-5479
Jens C. Pruessner	https://orcid.org/0000-0002-8582-2980
Eva Unternaehrer	https://orcid.org/0000-0002-3507-1883

Table of content

Participant characteristics

Table S1. Participant characteristics 3

Manipulation checks

Table S2. Glucose trajectories, model comparisons 4

Table S3. Arousal trajectories, model comparisons 5

Table S4. Pleasure trajectories, model comparisons 6

Effect of Drink and MC on cortisol trajectories during stress

Table S5. Cortisol trajectories, model comparisons 7

Table S6. Final cortisol model, parameters 8

Table S7. Full cortisol model, summary 9

Effect of Drink and MC on blood glucose trajectories

Table S8. Final glucose model, parameters 10

Effect of Drink and MC on alpha amylase trajectories during stress

Table S9. Alpha amylase, model comparisons 11

Table S10. Final alpha amylase model, parameters 12

Table S1. Participant characteristics

Table S1. Participant characteristics of the six experimental groups

	Drink						
	water			glucose			
MC	very high	high	low	very high	high	low	*p* [c]
Number	18	15	16	15	18	18	
Age [yr]	22.00(2.59)	21.67(1.80)	23.44(2.90)	22.27(3.10)	22.17(1.89)	22.33(2.85)	.557
BMI [kg/m^2]	21.23(1.85)	22.29(2.85)	21.81(1.89)	21.71(2.69)	20.81(2.18)	22.16(3.72)	.697
Hormonal status[a,b] (FP/LP/ OC)	13/0/5	11/0/4	8/0/8	8/0/7	12/0/5	14/1/3	.367
Time[a] (8 am/ 10 am)	10/8	9/6	12/4	10/5	5/13	11/7	.103
BDI	1.94(2.31)	4.73(4.74)	8.63(5.33)	3.40(3.68)	6.5(5.00)	6.17(3.94)	.001[d]
RSES	15.83(1.86)	15.00(2.07)	16.88(2.92)	15.67(2.23)	16.83(2.53)	16.17(2.18)	.182

Note. Mean values (± standard deviations) or absolute frequencies of participant characteristics. MC = maternal care, BMI = body mass index, FP = Follicular phase, LP = Luteal phase, OC = use of oral contraceptives, RSES = Rosenberg Self-Esteem Scale.

[a] at day of testing.

[b] due to missing values analysis depends on *n* = 99.

[c] p-values result from one-way ANOVA (for self-esteem), Kruskal-Wallis tests (for age, BMI, BDI) and Chi-square tests (for hormonal status, time).

[d] differences occur between the very high MC & water in comparison to the low MC & water, high MC & glucose and low MC & glucose groups.

Table S2. Glucose trajectories, model comparisons

Table S2. Comparison of growth curve models for glucose trajectories after step-wise incorporation of predictors using Analyses of Variance.

Model	number	Test	*df*	*logLikelihood*	χ^2	*p*
Baseline	1		2	-1355.61		
~ + random intercept	2	1 vs 2	3	-1349.21	12.81	<.001
random intercept + *Time*	3	2 vs 3	4	-1333.55	31.31	<.001
random intercept + $Time^2$	4	3 vs 4	5	-1305.47	56.16	<.001
~+ random $Time^2$	5	4 vs 5	13	-1258.22	94.50	<.001
~+ CAR1	6	5 vs 6	14	-1258.22	0.00	.989
random $Time^2$ + *Drink*	7	5 vs 7	14	-1251.44	13.56	<.001
~ + $Time^2$ x *Drink*	8	7 vs 8	16	-1203.18	96.51	<.001
~ + *MC*	9	8 vs 9	18	-1201.87	2.63	.269
~ + $Time^2$ x *MC*	10	9 vs 10	22	-1199.45	4.84	.305
~ + *Drink* x *MC*	11	10 vs 11	24	-1199.11	0.68	.712
~ + $Time^2$ x *Drink* x *MC*	12	11 vs 12	28	-1193.39	11.44	.022

Note. '~ +' indicates that the new model was built by adding the predictor following the tilde to the previous model. '+' indicates that the new model was built by adding a factor to the specified model. In the Baseline model fixed intercept constitutes the only predictor. *Time* represents the fixed linear, $Time^2$ the fixed quadratic effect of time. Random $Time^2$ represents the random quadratic effect of time. *Drink* was entered as dummy variable (water = 0, glucose = 1). $Time^2$ *x Drink* represents the model including the interaction between the quadratic effect of time and drink, *MC x Drink* represents the model including the interaction between maternal care and the quadratic effect of time, $Time^2$x *Drink x MC* the model including the three-way interaction between maternal care, drink and the quadratic effect of time. CAR1 = First-order autoregressive covariance structure.

Table S3. Arousal trajectories, model comparisons

Table S3. Comparison of growth curve models for arousal trajectories after step-wise incorporation of predictors using Analyses of Variance.

Model	number	Test	*df*	*logLikelihood*	χ^2	*p*
Baseline	1		2	-1378.21		
~ + random intercept	2	1 vs 2	3	-1359.89	36.65	<.001
random intercept + *Time*	3	2 vs 3	4	-1358.96	1.84	.174
random intercept +$Time^2$	4	3 vs 4	5	-1250.22	217.48	<.001
random intercept +$Time^3$	5	4 vs 5	6	-1248.91	2.63	.105
~ + random *Time*	6	4 vs 6	7	-1248.83	2.78	.249
~ + random $Time^2$	7	6 vs 7	13	-1243.10	11.47	.075
~ + CAR1	8	7 vs 8	14	-1240.29	5.63	.018

Note. '~ +' indicates that the new model was built by adding the predictor following the tilde to the previous model. '+' indicates that the new model was built by adding a factor to the specified model. In the Baseline model fixed intercept constitutes the only predictor. *Time* represents the fixed linear, $Time^2$ the fixed quadratic effect of time. Random $Time^2$ represents the random quadratic effect of time. CAR1=First-order autoregressive covariance structure.

Table S4. Pleasure trajectories, model comparisons

Table S4. Comparison of growth curve models for pleasure trajectories after step-wise incorporation of predictors using Analyses of Variance.

Model	number	Test	*df*	*logLikelihood*	χ^2	*p*
Baseline	1		2	-1510.97		
~ + random intercept	2	1 vs 2	3	-1449.11	123.72	<.001
random intercept + *Time*	3	2 vs 3	4	-1446.32	5.57	.018
random intercept + $Time^2$	4	3 vs 4	5	-1378.77	135.10	<.001
random intercept + $Time^3$	5	4 vs 5	6	-1376.99	3.57	.059
~ + random *Time*	6	4 vs 6	7	-1378.54	0.46	.795
~ + random $Time^2$	7	6 vs 7	13	-1371.73	13.63	.034
~ + CAR1	8	7 vs 8	14	-1361.42	20.61	<.001

Note. '~ +' indicates that the new model was built by adding the predictor following the tilde to the previous model. '+' indicates that the new model was built by adding a factor to the specified model. In the Baseline model fixed intercept constitutes the only predictor. *Time* represents the fixed linear, $Time^2$ the fixed quadratic effect of time. Random $Time^2$ represents the random quadratic effect of time. CAR1 = First-order autoregressive covariance structure.

Table S5. Cortisol trajectories, model comparisons

Table S5. Comparison of the growth curve models for cortisol trajectories after step-wise incorporation of predictors using Analyses of Variance.

Model	number	Test	*df*	*logLikelihood*	χ^2	*p*
Baseline	1		2	-704.00		
~ + random intercept	2	1 vs 2	3	-524.31	359.37	<.001
random intercept + *Time*	3	2 vs 3	4	-516.67	15.29	<.001
random intercept + $Time^2$	4	3 vs 4	5	-513.61	6.10	.013
random intercept + $Time^3$	5	4 vs 5	6	-511.15	4.93	.026
$Time^3$ + random *Time*	6	5 vs 6	8	-415.49	191.31	<.001
$Time^3$ + random $Time^2$	7	6 vs 7	14	-378.76	73.48	<.001
$Time^3$ + random $Time^3$	8	7 vs 8	24	-365.69	26.13	.004
~ + *Drink*	9	8 vs 9	25	-364.74	1.89	.169
~ + $Time^3$ x *Drink*	10	9 vs 10	28	-359.37	10.75	.013
~ + *MC*	11	10 vs 11	30	-359.20	0.35	.841
~ + $Time^3$ x *MC*	12	12 vs 11	36	-355.41	7.57	.271
~ + *Drink* x *MC*	13	13 vs 12	38	-355.31	0.20	.905
~ + $Time^3$ x *Drink* x *MC*	14	13 vs 14	44	-354.58	1.46	.962

Note. '~ +' indicates that the new model was built by adding the predictor following the tilde to the previous model. '+' indicates that the new model was built by adding a factor to the specified model. In the Baseline model fixed intercept constitutes the only predictor. *Time* represents the fixed linear, $Time^2$ the fixed quadratic and $Time^3$ the fixed cubic effect of time. Random *Time* represents the random linear effect of time. Random $Time^2$ represents the random quadratic effect of time. Random $Time^3$ represents the random cubic effect of time. *Drink* was entered as dummy variable (water = 0, glucose = 1). $Time^3$ *x Drink* represents the model including the interaction between the cubic effect of time and drink, $Time^3$ *x MC* represents the model including the interaction between the cubic effect of time and maternal care. *Drink x MC* represents the model including the interaction between Drink and maternal care. $Time^3$ x *Drink x MC* the model including the three-way interaction between maternal care, drink and the cubic effect of time.

Table S6. Final cortisol model, parameters

Table S6. Parameters of the final model for z-standardized cortisol levels during the stress response period.

	Coefficient (β) ± *SE*	*t(df)*	*p*
Intercept	-1.56(0.12)	-1.27(394)	.206
Time	0.40(1.20)	0.32(394)	.746
Time^2	-0.64(0.62)	-1.02(394)	.308
Time^3	-0.39(0.37)	-1.05(394)	.292
$\text{Drink}_{\text{water-glucose}}$	0.34(0.17)	1.98(98)	.050
Time x $\text{Drink}_{\text{water-glucose}}$	3.27(1.72)	1.90(394)	.058
Time^2 *x* $\text{Drink}_{\text{water-glucose}}$	-1.27(0.88)	-1.44(394)	.149
Time^3 *x* $\text{Drink}_{\text{water-glucose}}$	-1.49(0.52)	-2.87(394)	.004

Note. Parameters of the final Time^3 x Drink growth curve model for z-standardized cortisol trajectories. *Time* represents the linear, *Time*^{2} represents the quadratic and *Time*^{3} represents the cubic effect of time. *Drink* was entered as a dummy variable (water = 0, glucose = 1). Contrasts were defined by using water condition as reference group. $\text{Drink}_{\text{water-glucose}}$ represents the effect of drink. *Time x* $\text{Drink}_{\text{water-glucose}}$ represents the interaction between the linear effect of time and drink. *Time*^{2} *x* $\text{Drink}_{\text{water-glucose}}$ represents the interaction between the quadratic effect of time and drink. *Time*^{3} *x* $\text{Drink}_{\text{water-glucose}}$ represents the interaction between the cubic effect of time and drink.

Table S7. Full cortisol model, summary

Table S7. Summary of full model with *Time* (linear, quadratic, cubic), *Drink* and *MC* as predictors and z-standardized cortisol levels during the stress response period as outcome variable.

Effects	Statistic	*p*
Intercept	$F(1, 382) = 1.017$	0.314
$Time^3$	$F(3, 382) = 10.141$	<.001
Drink	$F(1, 94) = 2.011$	0.159
MC	$F(2, 94) = 0.165$	0.848
$Time^3$ *x Drink*	$F(3, 382) = 3.654$	0.013
$Time^3$ *x MC*	$F(6, 382) = 1.249$	0.281
Drink x MC	$F(2, 94) = 0.096$	0.908
$Time^3$ *x Drink x MC*	$F(6, 382) = 0.234$	0.965

Note. Effects of the full $Time^3$ x Drink x MC growth curve model for z-standardized cortisol trajectories. $Time^3$ represents the cubic effect of time. $Time^3$ *x Drink* represents the interaction between the cubic effect of time and Drink. $Time^3$ *x MC* represents the interaction between the cubic effect of time and maternal care. *Drink x MC* represents the interaction between Drink and maternal care. $Time^3$ *x Drink x MC* represents the three-way interaction between the cubic effect of time, Drink and maternal care.

Table S8. Final glucose model, parameters

Table S8 Parameters of the final model for blood glucose levels.

	Coefficient (b) ± *SE*	*t(df)*	*p*
Intercept	91.19(2.55)	35.77(188)	<.001
Time	15.57(27.95)	0.56(188)	0.578
$Time^2$	9.46(29.17)	0.32(188)	0.746
$Drink_{water\text{-}glucose}$	25.36(3.78)	6.71(94)	<.001
$MC_{very\ high\text{-}high}$	-1.84(3.78)	-0.49(94)	0.628
$MC_{very\ high\text{-}low}$	3.20(3.72)	0.86(94)	0.391
$Time \times Drink_{water\text{-}glucose}$	247.74(41.46)	5.98(188)	<.001
$Time^2 \times Drink_{water\text{-}glucose}$	-231.55(43.26)	-5.35(188)	<.001
$Time \times MC_{very\ high\text{-}high}$	36.79(41.45)	0.89(188)	0.376
$Time^2 \times MC_{very\ high\text{-}high}$	-18.00(43.26)	-0.42(188)	0.678
$Time \times MC_{very\ high\text{-}low}$	29.11(40.75)	0.71(188)	0.476
$Time^2 \times MC_{very\ high\text{-}low}$	-17.18(42.52)	-0.40(188)	0.687
$Drink_{water\text{-}glucose} \times MC_{very\ high\text{-}high}$	-3.04(5.35)	-0.57(94)	0.571
$Drink_{water\text{-}glucose} \times MC_{very\ high\text{-}low}$	-6.40(5.30)	-1.21(94)	0.230
$Time \times Drink_{water\text{-}glucose} \times MC_{very\ high\text{-}high}$	-145.99(58.63)	-2.49(188)	0.014
$Time^2 \times Drink_{water\text{-}glucose} \times MC_{very\ high\text{-}high}$	9.98(61.18)	0.16(188)	0.871
$Time \times Drink_{water\text{-}glucose} \times MC_{very\ high\text{-}low}$	-177.29(58.13)	-3.05(188)	0.003
$Time^2 \times Drink_{water\text{-}glucose} \times MC_{very\ high\text{-}low}$	-47.20(60.66)	-0.78(188)	0.438

Note. Parameters of the full $Time^2$ x Drink x MC growth curve model for blood glucose trajectories. *Time* represents the linear, $Time^2$ represents the quadratic effect of time. *Drink* was entered as a dummy variable (water = 0, glucose = 1). MC was entered as categorial variable (1 = very high, 2 = high, 3 = low). Contrasts were defined by using the water respectively the very high MC group as reference groups. $Drink_{water\text{-}glucose}$ represents the effect of drink. $MC_{very\ high\text{-}high}$ represents the difference between the high compared to the very high MC group. $MC_{very\ high\text{-}low}$ represents the difference between the low compared to the very high MC group.

Table S9. Alpha amylase, model comparisons

Table S9. Comparison of the growth curve models for alpha amylase trajectories after step-wise incorporation of predictors using Analyses of Variance.

Model	number	Test	*df*	*logLikelihood*	χ^2	*p*
Baseline	1		2	-2972.10		
~ + random intercept	2	1 vs 2	3	-2753.98	436.25	<.001
random intercept + *Time*	3	2 vs 3	4	-2747.17	13.62	<.001
random intercept + $Time^2$	4	3 vs 4	5	-2704.25	85.83	<.001
random intercept + $Time^3$	5	4 vs 5	6	-2678.32	51.86	<.001
$Time^3$ + random $Time^2$	6	5 vs 6	14	-2650.28	56.09	<.001
$Time^3$ + random $Time^3$	7	6 vs 7	24	-2607.31	85.93	<.001
~ + *Drink*	8	7 vs 8	25	-2606.97	0.68	0.409
~ + $Time^3$ x *Drink*	9	8 vs 9	28	-2605.92	2.11	0.550
~ + *MC*	10	9 vs 10	30	-2605.63	0.57	0.753
~ + $Time^3$ x *MC*	11	10 vs 11	36	-2599.89	11.48	0.075
~ + *Drink x MC*	12	11 vs 12	38	-2594.12	11.54	0.003
~ + $Time^3$ x *Drink x MC*	13	12 vs 13	44	-2592.29	3.66	0.722

Note. '~ +' indicates that the new model was built by adding the predictor following the tilde to the previous model. '+' indicates that the new model was built by adding a factor to the specified model. In the Baseline model fixed intercept constitutes the only predictor. *Time* represents the fixed linear, $Time^2$ the fixed quadratic and $Time^3$ the fixed cubic effect of time. Random $Time^2$ represents the random quadratic effect of time. Random $Time^3$ represents the random cubic effect of time. *Drink* was entered as dummy variable (water = 0, glucose = 1). $Time^3$ *x Drink* represents the model including the interaction between the cubic effect of time and drink, *MC x Drink* represents the model including the interaction between maternal care and the cubic effect of time, $Time^3$ x *Drink x MC* the model including the three-way interaction between maternal care, drink and the cubic effect of time.

Table S10. Final alpha amylase model, parameters

Table S10. Parameters of the final model for alpha amylase levels during stress.

	Coefficient (b) ± *SE*	*t(df)*	*p*
Intercept	99.63(18.47)	5.40(388)	<.001
Time	-229.47(67.66)	-3.39(388)	.001
$Time^2$	-289.90(84.70)	-3.42(388)	.001
$Time^3$	169.27(84.16)	2.01(388)	.045
$Drink_{water\text{-}glucose}$	6.00(24.94)	0.24(94)	.810
$MC_{very\ high\text{-}high}$	36.11(26.10)	1.38(94)	.170
$MC_{very\ high\text{-}low}$	-12.77(25.71)	-0.50(94)	.621
$Time \times Drink_{water\text{-}glucose}$	91.19(69.09)	1.32(388)	.188
$Time^2 \times Drink_{water\text{-}glucose}$	7.59(86.49)	0.09(388)	.930
$Time^3 \times Drink_{water\text{-}glucose}$	-45.21(85.93)	-0.53(388)	.599
$Time \times MC_{very\ high\text{-}high}$	-20.79(84.99)	-0.24(388)	.807
$Time^2 \times MC_{very\ high\text{-}high}$	-121.39(106.40)	-1.14(388)	.255
$Time^3 \times MC_{very\ high\text{-}high}$	246.64(105.72)	2.33(388)	.020
$Time \times MC_{very\ high\text{-}low}$	74.69(84.29)	0.89(388)	.376
$Time^2 \times MC_{very\ high\text{-}low}$	-182.64(105.52)	-1.73(388)	.084
$Time^3 \times MC_{very\ high\text{-}low}$	144.38(104.85)	1.38(388)	.169
$Drink_{water\text{-}glucose} \times MC_{very\ high\text{-}high}$	-56.22(31.82)	-1.77(94)	.081
$Drink_{water\text{-}glucose} \times MC_{very\ high\text{-}low}$	54.07(31.55)	1.71(94)	.090

Note. Parameters of the final Drink x MC growth curve model for alpha amylase trajectories. *Time* represents the linear, $Time^2$ represents the quadratic and $Time^3$ represents the cubic effect of time. *Drink* was entered as a dummy variable (water = 0, glucose = 1). MC was entered as categorial variable (1 = very high, 2 = high, 3 = low). Contrasts were defined by using the water respectively the very high MC group as reference groups. $Drink_{water\text{-}glucose}$ represents the effect of drink. $MC_{very\ high\text{-}high}$ represents the difference between the high compared to the very high MC group. $MC_{very\ high\text{-}low}$ represents the difference between the low compared to the very high MC group.
